# Vitamin D supplementation during pregnancy and the role of maternal prenatal depression

**DOI:** 10.1186/s12884-024-06631-8

**Published:** 2024-06-18

**Authors:** Bin lv, Ai Zheng, Ling Han

**Affiliations:** 1 Department of Obstetrics and Gynecology, West China Second Hospital, Sichuan University, Chengdu, China; 2 Key Laboratory of Birth defects and Related Diseases of Women and Children, Sichuan University, Ministry of Education, Chengdu, China; 3 Department of Gynecology and Obstetrics, West China Second hospital, Sichuan University, 3 Section of People South Street, Chengdu, 610041 P. R. China

**Keywords:** Vitamin D, Depression, Supplementation, Pregnancy

## Abstract

**Background:**

The current study sought to investigate the correlation between vitamin D supplementation in pregnant women with vitamin D deficiency in early pregnancy and the incidence of prenatal depression prior to delivery.

**Methods:**

This is a retrospective, single-center study that was conducted at a tertiary hospital in Chengdu, China. We conducted an analysis on pregnant women who were initially diagnosed with vitamin D deficiency at 12–14 weeks of gestation. After starting vitamin D supplementation at a dose of 800 IU daily from 14 weeks onwards, we measured both their vitamin D concentration and depression scores again during median gestational week 39 prior to delivery.

**Results:**

The study cohort comprised 1365 women who had been diagnosed with vitamin D deficiency at 12–14 weeks of gestation between November 1st, 2021 to November 1st, 2022. 537 pairs were matched based on a propensity score to control for other confounding factors. After propensity score matching, the baseline vitamin D levels were made consistent between the groups (*P* = 0.512). The incidence of depression in patients in vitamin D deficiency group following vitamin D supplementation was significantly higher than insufficiency group and reached statistical significance (*P* < 0.001). Additionally, we observed that serum 25-(OH) D concentration achieving insufficiency status after supplementation was 59.12%.

**Conclusion:**

Our study indicates that daily supplementation of 800IU of vitamin D can improve the depressive symptoms of individuals who are vitamin D deficiency during early pregnancy but achieve vitamin D insufficiency after supplementation during prenatal period.

## Background

Depression during pregnancy is a significant mental health disorder that arises from various psychological, physical, and social factors related to pregnancy. A systematic review indicates that the prevalence of perinatal depression is 11.9%, with higher rates observed in low-income countries [1]. A recent meta-analysis conducted in China has reported a prevalence of 19.7% for prenatal depression and 14.8% for postpartum depression [2]. Perinatal depression can have detrimental effects on both the mother and the baby, underscoring the importance of identifying its causes and implementing effective interventions. Increased attention should be directed towards perinatal depression in order to mitigate its impact.

In addition to the well-established risk factors such as genetic, psychological, physiological, and social factors, there has been growing attention towards the role of nutrition in perinatal depression [3]. Vitamin D, which is primarily synthesized in the skin upon exposure to sunlight, plays a crucial role in calcium and phosphate metabolism, impacting bone health. Its deficiency is a global concern and can also affect various other functions, including cell growth, neuromuscular function, and immune system regulation [4]. A meta-analysis conducted across 23 African countries, involving 21,474 participants, found the pooled prevalence of vitamin D deficiency to be 18.46% [5]. Additionally, a significant number of pregnant women have been reported to be deficient in vitamin D during the first trimester [6]. Vitamin D deficiency during pregnancy can have adverse effects on both maternal and fetal health, increasing the risk of pregnancy complications and maternal mental health issues. Therefore, addressing the issue of vitamin D deficiency during pregnancy is crucial in order to mitigate its negative impacts.

Several studies have explored the association between vitamin D deficiency and perinatal depression, with some reporting an elevated incidence of perinatal depression in individuals with deficient vitamin D levels [7, 8]. Moreover, prior research has suggested a positive correlation between vitamin D levels and mental health [9]. These findings imply that maintaining appropriate levels of vitamin D during pregnancy could potentially reduce the incidence of perinatal depression. Therefore, the current study aimed to investigate the relationship between vitamin D supplementation in pregnant women with vitamin D deficiency during early pregnancy and the incidence of prenatal depression before delivery.

## Methods

### Study population and data sources

This is a retrospective, observational single-center study conducted at a tertiary hospital in Chengdu, China (Registration number ChiCTR2200062225, Registration date July,30th). The study population included 1365 women who were diagnosed with vitamin D deficiency during the 12–14 weeks of gestation period between November 1st, 2021 to November 1st, 2022. Among them, 537 matches were obtained through propensity score matching to control for other potential confounding factors. The Ethics Committee of the West China Second University Hospital, Sichuan University provided approval for the study (2021 − 186).

This study conducted an analysis on pregnant women who were initially diagnosed with vitamin D deficiency during the 12–14 weeks of gestation. Subsequently, they received vitamin D supplementation at a dosage of 800 IU daily from 14 weeks onwards. We measured their vitamin D concentration and depression scores again between median gestational week 39 (38, 39) prior to delivery. General information was collected from all participants, including their age, BMI, number of abortion, gravity, parity, mode of conception, weight change, as well as any pregnancy-related comorbidities and complications. Furthermore, data on occupation, nationality, marital status, vitamin D levels, season, and depression index scores were recorded.

The inclusion criteria for this study were: (1) Normal depression score at gestational week 14. (2) Vitamin D deficiency was detected at gestational weeks 12–14. (3) Starting at gestational weeks 14, take 800 international units of vitamin D daily.

The exclusion criteria for this study were as follows: (1) Patients who were unwilling to participate or unable to complete follow-up. (2) Patients who do not take vitamin D supplements regularly. (3) have suffered from depression or other psychiatric mental illness. (4) emergency delivery.

To minimize selection bias, the cohort for this study was constructed by including all cases with complete records between November 1st, 2021 and November 1st, 2022. The vitamin D status of participants was determined by measuring the serum 25-hydroxyvitamin D (25(OH)D) concentration. According to The Institute of Medicine’s definition, serum 25(OH)D levels were categorized as deficiency (< 30nmol/L), insufficiency (30nmol/L-50nmol/L), or adequate (> 50nmol/L) [10]. All patients with vitamin D deficiency received indiscriminate vitamin D supplementation. Subsequently, using a threshold of 30nmol/L for vitamin D levels, patients were divided into insufficiency and deficiency groups, followed by further propensity score matching (PSM). The serum 25(OH)D concentration was measured using a chemiluminescence immunoassay analyzer (DiaSorin Inc., Stillwater, MN, USA). Prior to daily patient sample tests, indoor quality control tests were conducted. Bio-Rad third-party quality control products were used during patient sample testing to ensure internal quality control.

The Zong’s Self-rating Depression Scale, the Hamilton 17-item Depression Scale, and the Edinburgh Postnatal Depression Scale were used to evaluate the depression status of each participant. The diagnosis of prenatal depression was confirmed by two experienced senior psychiatrists using the Structured Clinical Interview for Diagnostic and Statistical Manual of Mental Disorders, Fifth Edition (DSM-V) and the Chinese Classification and Diagnostic Criteria of Mental Disorders, 3rd edition (CCMD-3).

The primary objective of this study was to determine the incidence of depression in pregnant women with vitamin D deficiency in comparison to the vitamin D insufficiency group after undergoing vitamin D supplementation before delivery. The secondary objective was to assess the proportion of pregnant women who achieved insufficiency status following daily supplementation with 800 IU of vitamin D before delivery.

### Statistical analysis

Directed acyclic graphs (DAGs) were designed based on previous research and clinical experience to gather covariates that might influence prenatal Vitamin D (VD) levels and antenatal depression. Based on all gathered covariates, propensity scores matching (PSM) were used to establish comparable cohorts. The nearest neighbor matching method was utilized, with a caliper parameter set at 0.1 and a matching ratio of 1:1. Histograms of propensity scores before and after matching were used to verify the consistency between the two cohorts after matching. We compared the demographic and clinical characteristics of pregnant women with vitamin D deficiency and those with vitamin D insufficiency(before and after PSM. We initially assessed the normality of all continuous variables (since the sample size was < 5000, we applied the Kolmogorov-Smirnov test). As all continuous variables did not conform to a normal distribution, we utilized non-parametric tests (Wilcoxon rank-sum test) for their statistical analysis. Categorical variables were presented as frequency and percentage and evaluated using the χ2 test or Fisher exact test as appropriate. All analyses were performed in R software version 4.1.3 (http://www.R-project.org/) and R package “tidyverse”, “MatchIt”, “openxlsx” were utilized. A *p*-value of less than 0.05 was considered statistically significant.)

## Results

### Patients characteristics

**Fig. 1:**
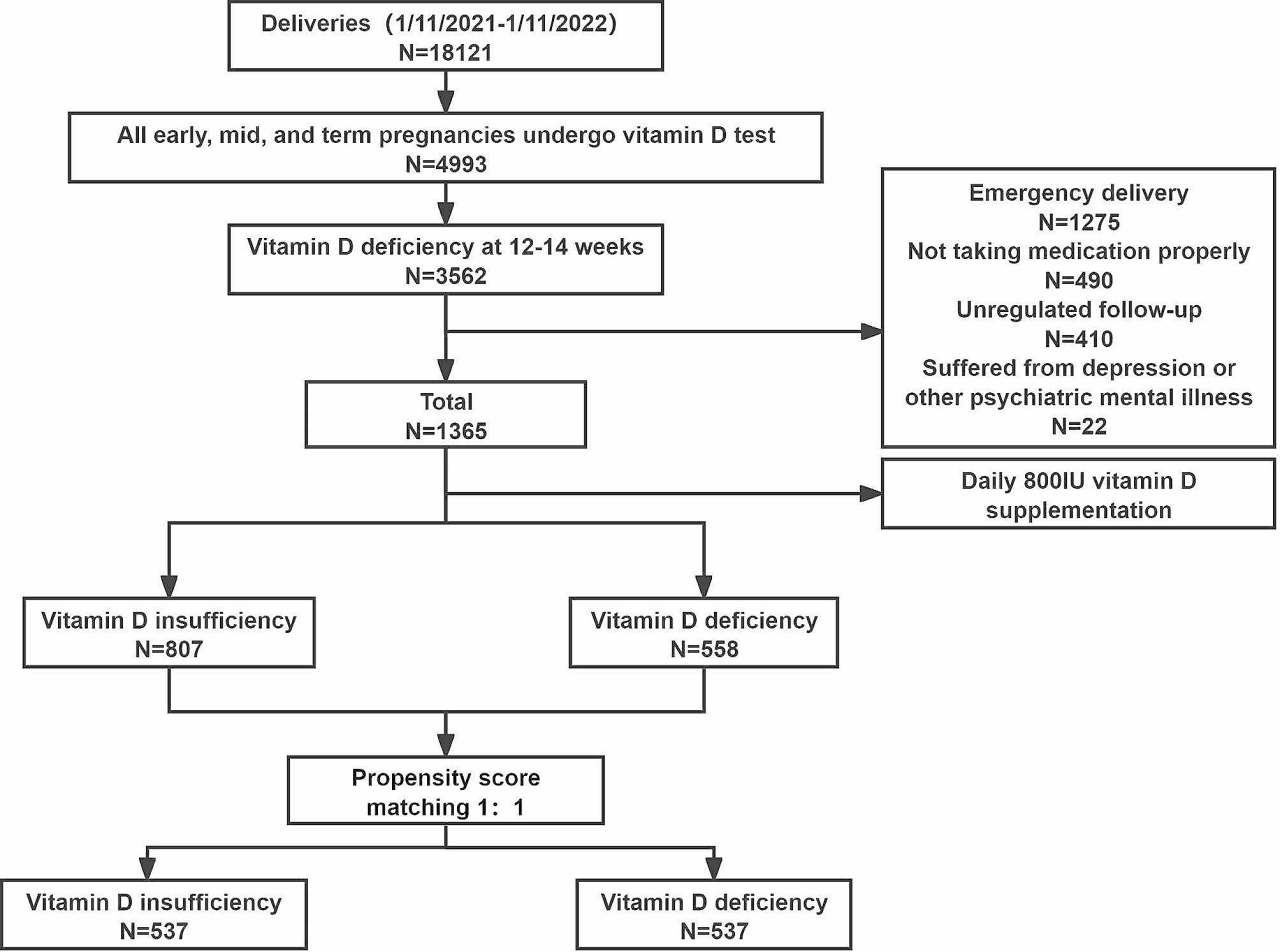
Flow diagram of included patients

During the specified period from November 1st, 2021 to November 1st, 2022, a total of 2,197 participants were excluded as they did not meet the predefined inclusion criteria. The detailed inclusion process is illustrated in Fig. 1. Eventually, a total of 1,365 patients were included in the study, with 807 patients in the vitamin D insufficiency group and 558 patients in the vitamin D deficiency group.

The patient characteristics, both before (Table 1) and after (Table 2) propensity score matching, are listed. The mean age of the vitamin D insufficiency group and vitamin D deficiency group were both 31 years, with no significant difference observed (*P* = 0.848). However, no other basic characteristics divulged any dissimilarities. Table 2 displays *p*-values exceeding 0.05 support the absence of statistical differences in all factors between the two groups except for vitamin D. The influence of other confounding factors in the two groups of data has been successfully excluded through the utilization of PSM. Subsequent to PSM, none of the basic characteristics were statistically different.

**Table 1:** The characteristics of patients before propensity score matching

	Before PSM		
	Insufficiency(*n* = 807)	Deficiency(*n* = 558)	*P*
Age, median (IQR)	31(28–34)	31(28–34)	0.645
Nationality, n (%)			0.537
Han	787(97.5%)	547(98.0%)	
Others	20(2.5%)	11(2.0%)	
Work status, n (%)			0.506
Employed	778(96.4%)	534(95.7%)	
Unemployed	29(3.6%)	24(4.3%)	
Only child, n (%)			0.082
Yes	758(93.9%)	536(96.1%)	
No	49(6.1%)	22(3.9%)	
Gravidity, n (%)	2(1–3)	2(1–3)	0.394
Parity, n (%)	0(0–1)	0(0–1)	0.593
Abortion, n (%)	1(0–1)	1(0–1)	0.573
BMI, median (IQR)	20.83(19.23–22.89)	20.83(19.38–22.94)	0.873
Gain weight (kg), median (IQR)	12.5(10–15)	12.5(10–15)	0.599
Weight(kg), median (IQR)	66.3(62–72)	66(61–73)	0.797
Poor marriage, n (%)			0.319
Yes	793(98.3%)	552(98.9%)	
No	14(1.7%)	6(1.1%)	
Pregnancy mode, n (%)			
Naturally	697(86.4%)	481(86.2%)	0.929
Assisted reproduction	110(13.6%)	77(13.8%)	
Diabetes, n (%)			
Yes	198(24.5%)	119(21.3%)	
No	609(75.5%)	439(78.7%)	0.167
Hypertension, n (%)			
Yes	26(3.2%)	17(3.0%)	
No	781(96.8%)	541(97.0%)	0.855
Heart disease, n (%)			
Yes	5(0.6%)	3(0.5%)	
No	802(99.4)	555(99.5%)	0.845
ICP, n (%)			
Yes	41(5.1%)	35(6.3%)	
No	766(94.9%)	523(93.7%)	0.345
Placenta previa, n (%)			
Yes	38(4.7%)	20(3.6%)	
No	769(95.3%)	538(96.4%)	0.311
Thyroid dysfunction, n (%)			
Yes	142(17.6%)	92(16.5%)	
No	665(82.4%)	466(83.5%)	0.593
Anemia, n (%)			
Yes	74(9.2%)	45(8.1%)	
No	733(90.8%)	513(91.9%)	0.477
Antiphospholipid syndrome, n (%)			
Yes	7(0.9%)	3(0.5%)	
No	800(99.1%)	555(99.5%)	0.540
Hepatitis B, n (%)			
Yes	49(6.1%)	28(5.0%)	
No	758(93.9%)	530(95.0%)	0.407
Chronic disease, n (%)			
Yes	9(1.1%)	2(0.4%)	
No	798(98.9%)	556(99.6%)	0.216
Twin pregnancies, n (%)			
Yes	50(6.2%)	27(4.8%)	
No	757(93.8%)	531(95.2%)	0.285
Fetal growth restriction, n (%)			
Yes	11(1.4%)	12(2.2%)	
No	796(98.6%)	546(97.8%)	0.266
Preterm birth, n (%)			
Yes	86(10.7%)	88(15.8%)	
No	721(89.3%)	470(84.2%)	0.005
Fetal malformation, n (%)			
Yes	8(1.0%)	3(0.5%)	
No	799(99.0%)	555(99.5%)	0.540
Stillbirth, n (%)			
Yes	1(0.1%)	2(0.4%)	
No	806(99.9%)	556(99.6%)	0.571
Season, n (%)			
Spring	136(16.9%)	92(16.5%)	0.566
Summer	176(21.8%)	138(24.7%)	
Autumn	259(32.1%)	164(29.4%)	
Winter	236(29.2%)	164(29.4%)	

**Table 2:** The characteristics of patients after propensity score matching

	After PSM(1:1)		
	Insufficiency(*n* = 537)	Deficiency(*n* = 537)	*P*
Age, median (IQR)	31(28-33.5)	31(28–34)	0.848
Nationality, n (%)			0.833
Han	525 (97.8%)	526 (98%)	
Others	12 (2.2%)	11 (2%)	
Work status, n (%)			0.878
Employed	514 (95.7%)	515 (95.9%)	
Unemployed	23 (4.3%)	22 (4.1%)	
Only child, n (%)			0.876
Yes	516 (96.1%)	515 (95.9%)	
No	21 (3.9%)	22 (4.1%)	
Gravidity, n (%)	2(1–3)	2(1–3)	0.448
Parity, n (%)	0(0–1)	0(0–1)	0.934
Abortion, n (%)	1(0–1)	1(0–1)	0.598
BMI, median (IQR)	20.83 (19.14, 22.65)	20.83 (19.38, 22.94)	0.314
Gain weight (kg), median (IQR)	13(10–15)	12.5(10–15)	0.975
Weight(kg), median (IQR)	53 (49, 59)	54 (49.5, 60)	0.292
Poor marriage, n (%)			1
Yes	528 (98.3%)	528 (98.3%)	
No	9 (1.7%)	9 (1.7%)	
Pregnancy mode, n (%)			0.362
Naturally	473 (88.1%)	463 (86.2%)	
Assisted reproduction	64 (11.9%)	74 (13.8%)	
Diabetes, n (%)			0.768
Yes	121 (22.5%)	117 (21.8%)	
No	416 (77.5%)	420 (78.2%)	
Hypertension, n (%)			1.000
Yes	17 (3.2%)	17 (3.2%)	
No	520 (96.8%)	520 (96.8%)	
Heart disease, n (%)			1.000
Yes	3 (0.6%)	3 (0.6%)	
No	534 (99.4%)	534 (99.4%)	
ICP, n (%)			0.708
Yes	36 (6.7%)	33 (6.1%)	
No	501 (93.3%)	504 (93.9%)	
Placenta previa, n (%)			0.750
Yes	22 (4.1%)	19 (3.5%)	
No	515(95.9%)	518(96.5%)	
Thyroid dysfunction, n (%)			0.687
Yes	96 (17.9%)	90 (16.8%)	
No	441(82.1%)	447(83.2%)	
Anemia, n (%)			0.567
Yes	39 (7.3%)	44 (8.2%)	
No	498 (92.7%)	493 (91.8%)	
Antiphospholipid syndrome, n (%)			1.000
Yes	3 (0.6%)	3 (0.6%)	
No	534 (99.4%)	534 (99.4%)	
Hepatitis B, n (%)			0.889
Yes	27 (5%)	28 (5.2%)	
No	510 (95%)	509 (94.8%)	
Chronic disease, n (%)			1.000
Yes	1 (0.2%)	2 (0.4%)	
No	536 (99.8%)	535 (99.6%)	
Twin pregnancies, n (%)			0.783
Yes	29 (5.4%)	27 (5%)	
No	508 (94.6%)	510 (95%)	
Fetal growth restriction, n (%)			0.247
Yes	7 (1.3%)	12 (2.2%)	
No	530 (98.7%)	525 (97.8%)	
Preterm birth, n (%)			0.370
Yes	67 (12.5%)	77 (14.3%)	
No	470 (87.5%)	460 (85.7%)	
Fetal malformation, n (%)			
Yes	3(0.5%)	3(0.5%)	1.000
No	534 (99.4%)	534 (99.4%)	
Stillbirth, n (%)			1.000
Yes	1(0.2%)	2(0.4%)	
No	536(99.8%)	535(99.6%)	
Season, n (%)			0.603
Spring	95 (17.7%)	89 (16.6%)	
Summer	112 (20.9%)	130 (24.2%)	
Autumn	168 (31.3%)	158 (29.4%)	
Winter	162 (30.1%)	160 (29.8%)	

### The depression outcome

Table 3 displays the depression outcomes following vitamin D supplementation. The baseline was consistent between the two groups, there was no statistical difference between the two groups before supplementation (*P* = 0.512), and there was a statistical difference in depression between the two groups after vitamin D supplementation(*P* = 1.197E-04).

**Table 3:** The 25(OH)D concentration and Depress status before and after propensity score matching

	Before PSM			After PSM(1:1)		
	Insufficiency(*n* = 807)	Deficiency (*n* = 558)	*P*	Insufficiency (*n* = 537)	Deficiency (*n* = 537)	*P*
25(OH)D(Before), median (IQR)	21(15.2–25.5)	17.9(13.1–23)	6.737E-09	17.65(12.8–21.6)	17.9(13.1–23)	0.512
25(OH)D(After), median (IQR)	39.3(34.5–44.3)	26.7(22.3–28.3)	1.029E-216	39.4(34.5-44.25)	26.7(22.35–28.35)	1.367E-176
Depression, n (%)			4.781E-08			1.197E-04
Yes	197(24.4%)	214(38.4%)		144(26.8%)	204(38.0%)	
No	610(75.6%)	344(61.6%)		393(73.2%)	333(62.0%)	

### Vitamin D supplementation result

Table 3 presents the results of daily supplementation with 800IU of vitamin D. Out of the 1365 cases, 807 cases were classified as vitamin D insufficiency, while 558 cases remained deficient in vitamin D. The proportion of cases that serum 25-(OH) D concentration achieving insufficiency status after supplementation was 59.12%.

## Discussion

Our study has indicated that daily supplementation of 800 IU vitamin D starting at 14 weeks of pregnancy can alleviate depressive symptoms in individuals who are vitamin deficient during early pregnancy but attain vitamin D insufficiency after taking prenatal supplementation. Moreover, our findings suggest that 59.12% of cases achieved insufficiency status in serum 25-(OH) D concentration after such supplementation.

Our study has revealed a strong statistical significance in the prevalence of depression among pregnant women during the late trimester before delivery between those in the deficiency group versus those in the insufficiency group who received daily supplementation. These findings highlight the crucial role of vitamin D supplementation for pregnant women residing in areas with a high prevalence of vitamin D deficiency, and its potential impact on maternal health.

Vitamin D deficiency during the first trimester of pregnancy is a widespread concern globally. Choi et al. reported that the overall incidence of vitamin D deficiency and severe vitamin D deficiency in pregnant women was 77.3% and 28.6% respectively, with a higher prevalence observed in the first trimester among Korean women [11]. Similarly, Zhao et al. conducted a study involving 27,166 healthy pregnant women in China and found a pooled prevalence of vitamin D deficiency of 34.3%, with incidence rates of 37.83%, 33.8%, and 29.3% for the first, second, and third trimesters respectively [12]. Our study found that following daily supplementation of 800IU vitamin D, 59.12% of the women reached vitamin D insufficiency status. These results highlight the importance of vitamin D supplementation not only for the health of the fetus but also for maternal well-being. However, there is currently no consensus on the recommended daily intake of vitamin D during pregnancy. The Institute of Medicine and Health Canada currently recommend a daily vitamin D intake of 600 IU during pregnancy [13, 14]. While the American College of Obstetricians and Gynecologists (ACOG) suggest that 1,000–2,000 international units per day of vitamin D is safe, they also acknowledge that routine use requires verification through large randomized trials [15]. However, the World Health Organization (WHO) does not recommend routine supplementation of vitamin D [16]. These varying recommendations indicate the need for further research to elucidate the optimal dosage of vitamin D supplementation during pregnancy.

Our study confirmed that routine vitamin D supplementation during the first trimester can reduce the incidence of depression in pregnant women with vitamin D insufficiency before delivery. Although previous studies have shown a positive relationship between vitamin D supplementation and the reduction of depression, the evidence is inconclusive. A meta-analysis of 13 studies found that 12 of them reported a positive relationship between vitamin D supplementation and depression reduction in non-pregnant individuals [4]. Few studies have looked at the effect of vitamin D supplementation on perinatal depression. Vaziri et al. conducted a randomized trial in pregnant women with no history of mental illness and found that daily supplementation of 2000 IU vitamin D3 during late pregnancy reduced the incidence of perinatal depression [17]. Additionally, vitamin D supplementation has been found to improve motor functions in infants born to mothers with perinatal depression [18]. Our study specifically looked at the effect of routine supplementation on prenatal maternal depression in the vitamin D sufficiency group. Due to the varied outcomes of vitamin D supplementation in previous studies with non-pregnant individuals and the limited research on vitamin D supplementation during pregnancy, further clinical trials are warranted to validate these findings.

## Strengths and limitations

Our study has demonstrated that daily supplementation of vitamin D can improve depression status in pregnant women who were deficient in vitamin D during early pregnancy and had reached an insufficient serum 25-(OH) D concentration during the late trimester before delivery. These findings provide substantial evidence showcasing the importance of vitamin D supplementation for pregnant women, especially in regions where vitamin D sufficiency is not typically observed, and highlight the potential of vitamin D in promoting maternal health. However, as our study was retrospective in nature, there are potential limitations and biases that must be acknowledged. Selection bias and recall bias are possible influences on our results due to the retrospective design. Additionally, the study population consisted of participants from a research center, which may not be wholly representative of the wider population.

## Conclusions

Our study has indicated that daily supplementation of 800 IU of vitamin D can improve depressive symptoms during the prenatal period in individuals who are deficient in vitamin D during early pregnancy but achieve vitamin D insufficiency after undergoing prenatal supplementation. Additionally, we found that this supplementation protocol led to a 59.12% success rate in achieving insufficiency status during late pregnancy. Based on our results, we recommend that future research focus on identifying the appropriate dosage of vitamin D supplementation for pregnant women through well-planned multicenter randomized controlled trials.

## Data Availability

The original contributions presented in the study are included in the article/supplementary material, further inquiries can be directed to the corresponding authors.
